# Therapeutic Targets and Mechanism of Xingpi Jieyu Decoction in Depression: A Network Pharmacology Study

**DOI:** 10.1155/2021/5516525

**Published:** 2021-06-23

**Authors:** Ze Chang, Li-Juan He, Dang-Feng Tian, Qiang Gao, Jing-Feng Ling, Yu-Chun Wang, Zhen-Yun Han, Rong-Juan Guo

**Affiliations:** ^1^Dongfang Hospital of Beijing University of Chinese Medicine, Beijing 100000, China; ^2^Beijing University of Chinese Medicine, Beijing 100000, China; ^3^Shenzhen Hospital of Beijing University of Chinese Medicine, Shenzhen 518000, Guangdong, China

## Abstract

**Background:**

Depression is a common mental disease that lacks effective therapeutic drugs with good curative effects and few adverse reactions. Traditional Chinese medicine (TCM) has the advantages of multiple components, multiple channels, and fewer adverse reactions in the treatment of depression. Although Xingpi Jieyu Decoction (XPJYD) demonstrates a good therapeutic effect on depression, the pharmacological mechanism underlying its antidepressant effect is still unclear.

**Methods:**

We used a network pharmacology strategy, including the construction and analysis of a complex drug-disease network, to explore the complex mechanism of XPJYD treatment of depression. In addition, molecular docking technology was used to preliminarily study the binding ability of the potential active components and core therapeutic targets of XPJYD.

**Results:**

The network pharmacology results showed 42 targets of XPJYD that are involved in depression. PPI network analysis demonstrated that the top 10 core targets were AKT1, VEGFA, MAPK8, FOS, ESR1, NR3C1, IL6, HIF1A, NOS3, and AR. The molecular docking results showed that the binding energies of beta sitosterol with AR, FOS, AKT1, VEGFA, NR3C1, and NOS3 were less than −7.0 kcal·mol^−1^, indicating a good docking effect. The GO enrichment analysis results showed that the XPJYD antidepression mechanism mainly involves the following biological processes such as apoptotic signaling pathway, cellular response to lipid, inflammatory response, and others. The KEGG analysis results indicated that XPJYD may regulate 13 pathways such as PI3K-Akt signaling pathway and estrogen signaling pathway in the treatment of depression.

**Conclusions:**

This study reflects the characteristics of the mechanism of action by which XPJYD treats depression, which includes multiple components, multiple targets, and multiple pathways, and provides a biological basis for further verification and a novel perspective for drug discovery in depression.

## 1. Introduction

Depression (F32.900) is a kind of mental disease with obvious and lasting symptoms, such as low mood, decreased interest, and impaired cognitive function, which can affect a person's thoughts, behavior, feelings, and sense of well-being [1]. It has the characteristics of a high prevalence rate, high recurrence rate, high disability rate, and high suicide rate, which cause substantial burdens to patients, their families, and society [2–4]. Although depression will become the second largest disease in the world after heart disease, the pathogenesis of depression is complex and unclear [5]. Current research shows that there are several hypotheses for the pathogenesis of depression, including the monoamine neurotransmitter and receptor hypotheses, the hypothalamic-pituitary-adrenal (HPA) axis hypothesis, and the neurotrophic factor hypothesis, as well as hypotheses involving oxidative stress and neuroinflammation, synaptic plasticity, and intestinal microflora imbalance [6–9]. Currently, the most common clinical treatment for depression is the administration of SSRI and SNRI drugs [10]. However, such drugs have the disadvantages of slow onset of treatment, incomplete relief of symptoms, and numerous adverse reactions, leading to reduced compliance of patients with medication and incomplete treatment of depression [11]. The treatment of depression with Traditional Chinese medicine, which incorporates the perspectives of physical and mental adjustment and has multicomponent, multitarget, and multimechanism characteristics, leads to an individualized diagnosis and treatment program combining differentiation and treatment that has few adverse reactions and can effectively alleviate cognitive dysfunction and somatization symptoms associated with depression [12–14].

Traditional Chinese herbs such as Herbahypericiperforati, Acoritataninowii Rhizoma, Turmeric, and Panax quinquefolium have better antidepressant effects. Compared with selective 5-HT reuptake inhibitor (SSRI) antidepressants, Herba Hyperici perforati showed better efficacy and higher safety in the treatment of mild to moderate depression [15]. Some studies have found that *β*-asarum, the main component of Acoritataninowii Rhizoma, had a protective effect on hippocampal neurons, increased the phosphorylation levels of ERK1/2, and CREB in the hippocampus, and improved the expression of brain-derived neurotrophic factor BDNF in depressed rats [16, 17].

Xingpi Jieyu Decoction (XPJYD) is composed of Panacis Quinquefolii Radix, Herbahypericiperforati, Acoritataninowii Rhizoma, and Curcumae Radix. Previous studies have shown that XPJYD significantly decreased the preference for sugar water and the open-field test movement distance of depressed rats with CUMS and improved depressed behavior in depressed rats. Moreover, it increased the serum corticosterone concentration and reversed the serum 5-HT content in rats with depression, which is similar to the effect of sertraline, an SSRI antidepressant [18]. XPJYD is more effective than sertraline in improving learning and memory [19]. Depressed rats induced by chronic unpredictable mild stress showed oxidative stress injury in the hippocampus, cerebral cortex, skeletal muscle, and small intestine with disrupted mitochondrial function and energy metabolism in the later stage of stress [20, 21]. XPJYD can improve the function and energy metabolism of mitochondria in the hippocampus and cerebral cortex, and other regions of the brain and has an antioxidative stress effect that is better than that of sertraline [22]. In general, studies have shown that XPJYD may have antidepressant effects by inhibiting the HPA axis, increasing monoamine neurotransmitters such as 5-HT, and regulating mitochondrial function.

Clinical evidence shows that, compared with traditional Chinese medicine placebo, XPJYD can reduce the scores of HAMD-24, SDS, and PHQ-15, improve the depressive mood and somatic symptoms of patients with depression, and increase the level of ATP in peripheral blood [23]. Intestinal microecological imbalance, which can cause impaired intestinal mucosal barrier function, is closely related to the inflammatory response of depression. Lipopolysaccharide (LPS) can enter the circulation through the damaged intestinal mucosal barrier and induce a systemic inflammatory response, leaving depressed patients in a state of chronic low-grade inflammation [24, 25]. Small sample clinical studies have shown that XPJYD can reduce serum LPS, D-lactic acid, DAO content in patients with depression and then reduce serum IL-6 and TNF-a content, indicating that XPJYD can repair the intestinal mucosal barrier and reduce the inflammatory response to a certain extent [26].

**Figure 1:**
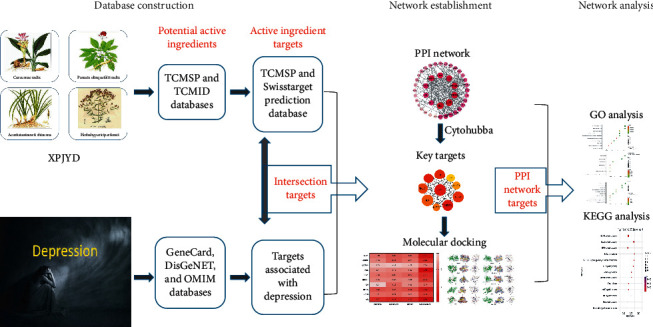
Flow chart of the research process in this study.

The research of network pharmacology on disease embodies the concept of systematicness, wholeness, and network, which is similar to the concept of wholeness in traditional Chinese medicine [27, 28]. Network pharmacology can explain, to a certain extent, the mechanism of action of multicomponent, multitarget, and multipathway of TCM compounds in the treatment of diseases [29]. Although XPJYD demonstrates a good therapeutic effect on depression, the pharmacological mechanism underlying its antidepressant effect is still unclear. Due to the complex composition of XPJYD, in this study, the network pharmacology method was used to study this Traditional Chinese medicine compound. By constructing a multilevel complex network of “disease-gene-target-drug” interactions, the potential material basis and multipathway mechanism of XPJYD in treating depression were explored, which provided a theoretical basis for its clinical application. The overall workflow of the study is shown in Figure 1.

## 2. Materials and Methods

### 2.1. Collection of Chemical Ingredients and Targets of XPJYD

All the components of XPJYD (American ginseng, *Hypericum perforatum*, Acorus gramineus, and Curcuma aromatica) were obtained by searching the Traditional Chinese Medicine Systems Pharmacology Database [30] (TCMSP) and Traditional Chinese Medicine Integrated Database [31] (TCMID). The potential active ingredients of XPJYD were screened by the following characteristics: standard oral bioavailability (OB) ≥30% and drug-like property (DL) ≥0.18 [32, 33]. We searched the targets of the potential active components of XPJYD in TCMSP and converted the gene names in the UniProt database. SwissTargetPrediction, which is an internet-based service for target prediction of bioactive small molecules, was used to make similarity predictions of the SMILES structure of each potential pharmacophore with *Homo sapiens* selected as the default species to predict the targets of potential drug molecules [34].

### 2.2. Acquisition of Targets Associated with Depression

Potential targets associated with depression were collected from the GeneCard [35], DisGeNET, and OMIM databases. The GeneCard database establishes the correlation ranking of genes and diseases and provides the Gifts algorithm [36]. Based on the relevance score, targets with higher Relevance can be further selected from many depression-related targets. By intersecting XPJYD component targets with disease targets, we obtained common targets for both drugs and diseases. Cytoscape software [37] was used to map the drug-component-target-disease network.

### 2.3. Construction of Protein-Protein Interaction Network

The shared targets between the drug targets and the disease targets were imported into the Search Tool for the Retrieval of Interacting Genes/Proteins (STRING) [38]. PPI network results were saved in TSV format and imported into Cytoscape3.7.1 software. The maximal clique centrality (MMC) algorithm in the cytoHubba plug-in was used for a comprehensive analysis of network topology to obtain the top 10 hub genes [39].

### 2.4. Gene Ontology and Pathway Enrichment Analysis

Gene Ontology (GO) enrichment analysis for overlapping genes was performed using the MetScape database (https://david.ncifcrf.gov/) to identify biological processes. The KOBAS database (http://kobas.cbi.pku.edu.cn/) [40] was used for KEGG pathway enrichment analysis with the “Human” setting to systematically explore pathways associated with the shared targets.

### 2.5. Molecular Docking

The PDB format of the core target protein was downloaded from the RCSB PDB database (http://www.rcsb.org/). We used PyMol 2.4 software to remove water molecules and separate the original ligand from the core target protein. We imported the processed protein targets into AutoDock Tools 1.5.6 software [41] to hydrogenate, calculate the total charge, and set the atomic type, and saved the files in the PDBQT format. The mol2 structures of the corresponding components of the core target were downloaded from the TCMSP database, and AutoDock Tools was used to set the rotatable bonds and to save the files in the PDBQT format. AutoDock-Vina software was used to perform molecular docking. Finally, PyMol and LigPlot + V.2.2 software were used to visualize the docking results and establish the docking interaction pattern.

## 3. Results

### 3.1. Acquisition of the Active Components and Targets of XPJYD

After screening with the set standards of OB ≥30% and DL ≥0.18, 33 potentially active ingredients were retrieved from TCMSP and TCMID, among which 7 were potential active ingredients of Hyacinth chrysoides, 11 were potential active ingredients of Panax quinquefolium, 4 were potential active ingredients of Acorus tatarinowii, and 15 were potential active ingredients of turmeric. Ginsenoside rh2 and its metabolites can reduce the activity duration of forced swimming test (FST) and tail suspension test (TST) in mice and improve the depression-like behavior induced by lipopolysaccharide (LPS), showing the highest antidepressant effect at 30 mg/kg [42]. Kaempferol alleviated hippocampal neuron injury in CUMS depression model rats by inhibiting autophagy and oxidative stress [43]. Quercetin alleviates LPS-induced depression-like behavior and learning and memory impairment in rats by regulating BDNF-related imbalance of Copine 6 and TREM1/2 expression in the hippocampus [44]. Naringenin improved depressive behavior and the learning and memory ability of rats with CUMS depression [45].

The corresponding target proteins of the active components were obtained by using TCMSP and SwissTargetPrediction, and the SwissTargetPrediction results were obtained according to a probability value above 0.5. Finally, 213 target proteins corresponding to 18 potentially active ingredients were acquired. The specific information of OB and DL values of 18 components is shown in Table 1.

### 3.2. Construction of the Drug-Component-Target-Disease Network

**Figure 2:**
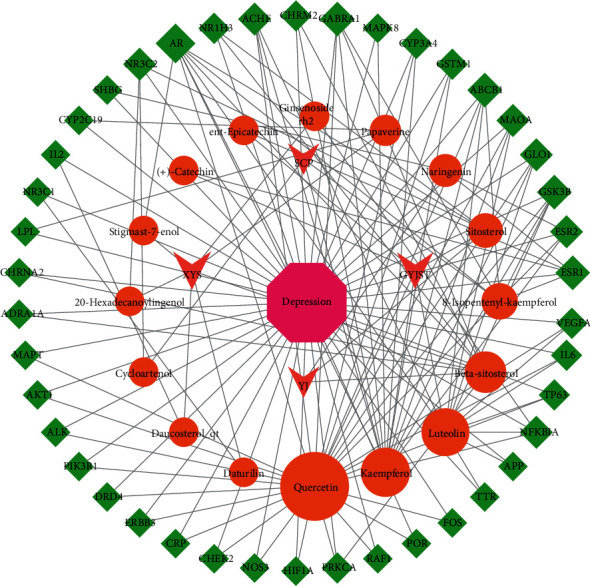
The drug-component-target-disease network diagram of XPJYD for the treatment of depression. Rhomboids represent the targets, and circle represents the active ingredients.

After screening with relevance score greater than 0.2 in GeneCard and score greater than 0.2 in DisGeNET, a total of 942 depression-related targets were retrieved from the GeneCard, OMIM, and DisGeNET databases. After the intersection of drug targets and disease targets, 42 drug-disease intersection targets were obtained, namely, the targets of XPJYD involved in the treatment of depression. Cytoscape3.7.1 software was used to construct the drug-component-target-disease network, and the results are shown in Figure 2. The figure shows that an active ingredient in XPJYD can correspond to multiple targets, and a target can also correspond to different active ingredients. The same disease may correspond to different targets, and different targets may correspond to different active components of XPJYD. These results indicated that the antidepressant mechanism of XPJYD has multicomponent and multitarget characteristics. The components with a degree value greater than 20 in the network topology analysis were quercetin (degree = 58), kaempferol (degree = 52), luteolin (degree = 26), and beta sitosterol (degree = 28).

### 3.3. PPI Network Analysis

**Figure 3:**
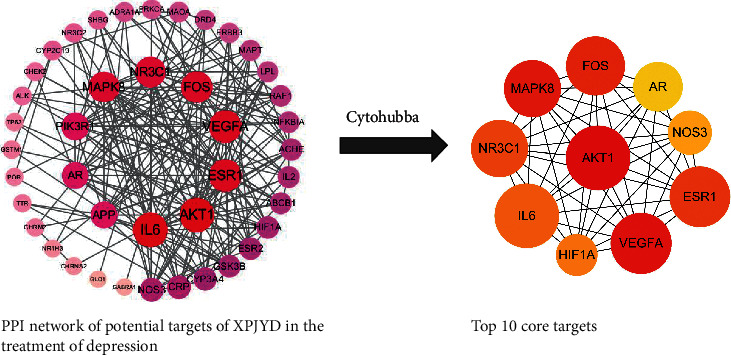
Identification of the top 10 hub genes in the PPI network.

The overlapping target genes were imported into the STRING database with medium confidence set to 0.4 to obtain a PPI network for the targets. The PPI network results were saved in TSV text format and imported into Cytoscape3.7.1 software for network topology analysis to obtain the top 10 hub genes. The results of the network topology analysis are shown in Figure 3. The PPI network contains 42 nodes with 207 edges, in which the circular nodes represent the target proteins, each edge represents the interaction relationship between the target protein and another protein, and the thickness of the edge represents the strength of the interaction force. The top 10 targets obtained on cytoHubba are AKT1, VEGFA, MAPK8, FOS, ESR1, NR3C1, IL6, HIF1A, NOS3, and AR. These targets play an important role in the PPI network, suggesting that they are important targets in the treatment of depression by XPJYD. There are three subtypes of AKT (AKT1, AKT2, and AKT3), and AKT1 is the most important subtype, which plays an important role in depression [46]. The study showed that AKT protein activity was significantly reduced in the brain tissues of suicidal patients with major depression [47]. Akt may enhance the efficacy of antidepressant drugs by enhancing the function of hippocampal stem cells [48]. AKT1 gene polymorphism was associated with treatment response in depressed patients [49]. Vascular endothelial growth factor (VEGF) plays an important regulatory role in inducing endothelial cell proliferation, promoting cell migration, inhibiting cell apoptosis, and inducing vascular permeability [50]. As a multifunctional angiogenic factor, it plays an important role in the treatment of depression [51]. Previous studies found that the expression levels of VEGFA in rats with chronic unpredictable mild stress-induced depression were significantly lower than those in the blank group [52]. A relevant meta-analysis showed that the concentrations of inflammatory factors such as IL-6 and TNF were higher than normal in patients with depression [53]. Estrogen receptors are divided into nuclear receptors and membrane receptors. Nuclear receptors include estrogen receptor *α* (ER*α*) and estrogen receptor *β* (ER*β*), while the main membrane receptor of estrogen is the G-protein-coupled estrogen receptor (GPER) [54]. It was found that the RS9340799 polymorphism of the ER gene may influence the development and outcome of depression [55]. ER*β* agonists were also found to reverse depression-like behavior in ovariectomized rats [56]. The GPER agonist G1 alleviated the depressive behavior of depressed rats by increasing GPER in the hippocampus [57]. Androgen receptor (AR) may influence the development of major depressive disorder [58]. The study suggests that AR deficiency may accelerate the development of depression-like behavior in chronically mildly stressed mice [59]. The mechanism may be to regulate the expression of brain-derived neurotrophic factor (BDNF) by altering the expression of miR-204-5p [60], thereby affecting the depression-like behavior of CMS mice.

### 3.4. Analysis of Molecular Docking Results

**Figure 4:**
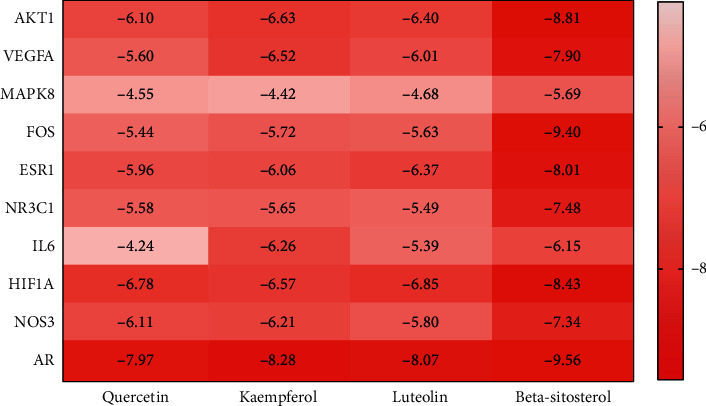
Heat map of molecular docking results.

Quercetin, kaempferol, luteolin, and beta sitosterol were used for molecular docking with the top 10 hub genes. The molecular docking results are presented in the form of a heat map (Figure 4). A binding energy (affinity) < −7.0 kcal·mol^−1^ indicates good binding activity. Lower binding energy indicates a better docking efficiency. The molecular docking results showed that the binding energies of beta sitosterol with AR, FOS, AKT1, VEGFA, NR3C1, and NOS3 were less than −7.0 kcal·mol^−1^, indicating a good docking effect. Kaempferol, luteolin, and quercetin have a good docking effect with AR genes.

**Figure 5:**
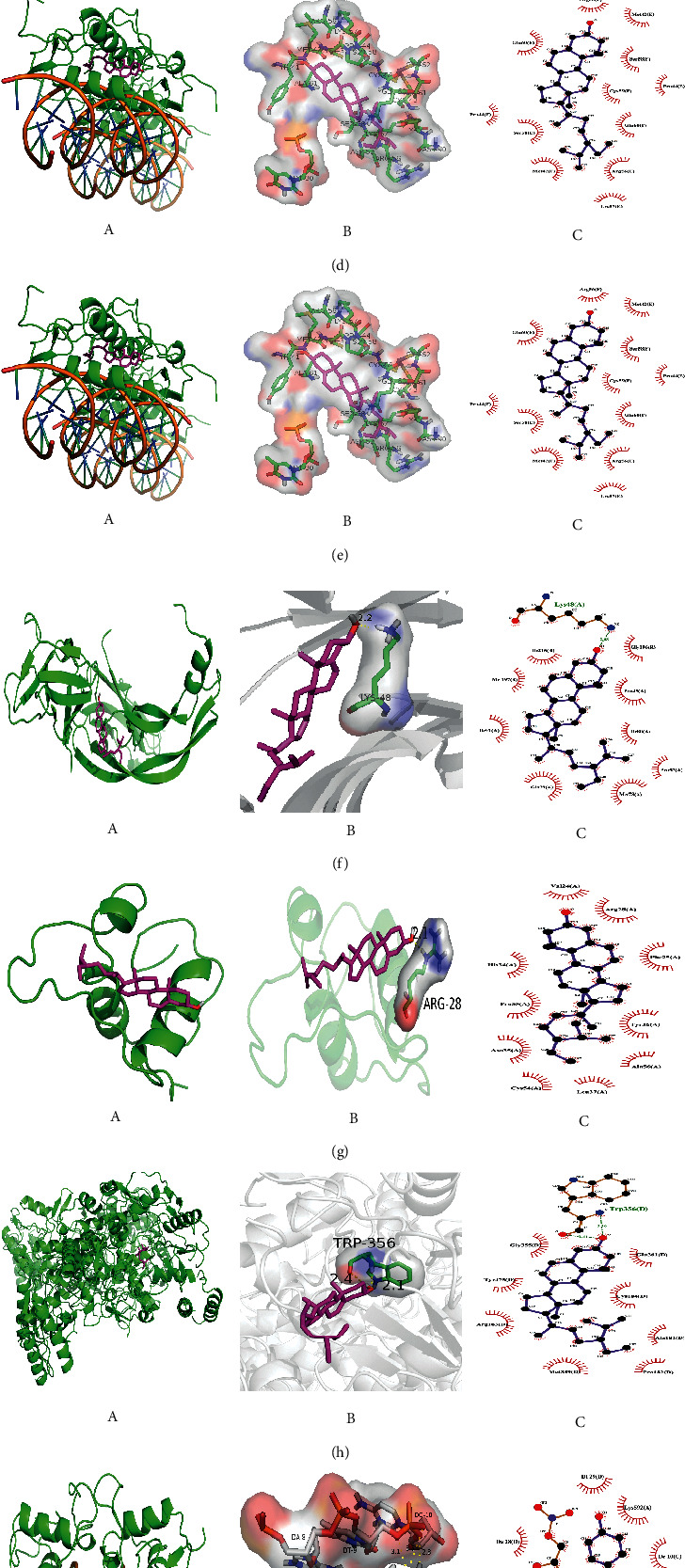
Schematic diagram of molecular docking results in less than −7.0 kcal·mol^−1^. (A) Overall map of binding sites of small molecules to proteins. (B) Detailed 3D diagrams. The small molecules are represented in yellow STICKS models. (C) Two-dimensional graphs of docking results. (a) Beta-sitosterol and AR, (b) beta sitosterol and FOS, (c) beta sitosterol and AKT1, (d) beta sitosterol and H1F1A, (e) beta sitosterol and ESR1, (f) beta sitosterol and VEGFA, (g) beta sitosterol and NR3C1, (h) beta sitosterol and NOS3, (i) kaempferol and AR, (j) quercetin and AR, (k) luteolin and AR.

Diagrams of the interaction structures of the interaction between the active ingredient and the hub genes with good binding energy were drawn. The diagrams are shown in Figure 5 and include 3 different representations designated A, B, and C. Representation A shows the binding sites of small molecules on the respective proteins. Representation B shows the detailed interactions between the small molecule compounds and the key residues on the respective proteins to show whether the small molecules and proteins interact at specific spatial locations. Representation C is a two-dimensional schematic for observing the hydrogen bond and hydrophobic action caused by small molecules and protein residues. The active ingredients such as beta sitosterol, kaempferol, quercetin, and luteolin form hydrogen bonds with AR, respectively. The results showed that beta sitosterol formed hydrogen bonds with FOS, VEGFA, NR3C1, and NOS3 genes.

### 3.5. GO Enrichment Analysis of the Overlapping Targets

**Figure 6:**
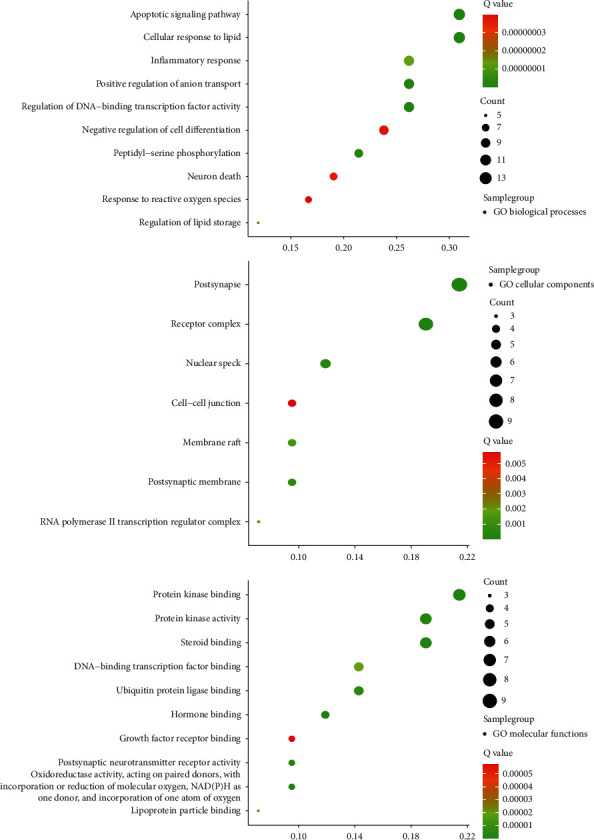
Results of GO enrichment analysis of the antidepressant targets.

GO enrichment analysis mainly involves three aspects: cell composition, molecular function, and biological process. As shown in Figure 6, the biological functions of the targets were analyzed using the Metscape database. The overlapping targets are mainly distributed in cell components such as postsynapse, receptor complex, and nuclear speck. and are involved in molecular functions such as steroid binding, hormone binding, and protein kinase binding. The results indicated that the antidepression mechanism of XPJYD mainly involves biological processes such as apoptotic signaling pathway, cellular response to lipid, inflammatory response, neuron death, response to reactive oxygen species, and others.

### 3.6. KEGG Analysis

**Figure 7:**
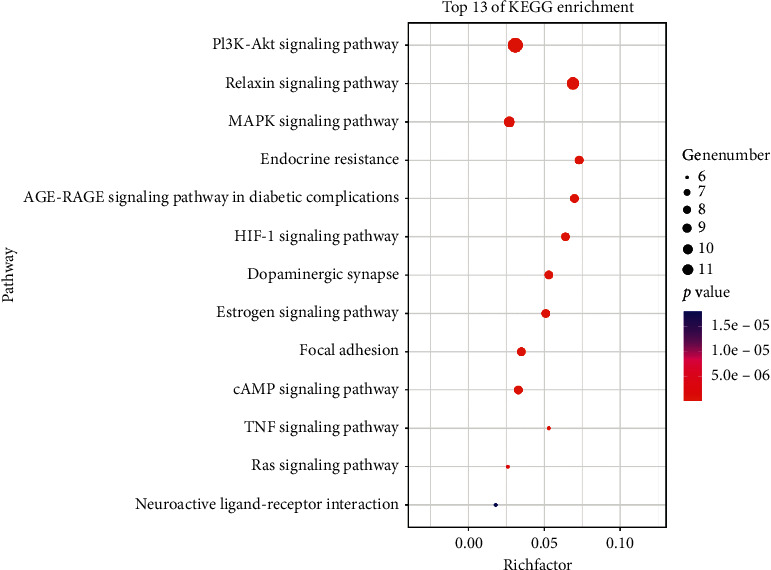
Bubble diagram of 13 pathways associated with depression.

We used the KOBAS database to analyze the KEGG pathways of the overlapping targets. A total of 198 pathways were enriched, of which the 13 pathways associated with depression are shown in Figure 7. The results showed that XPJYD may achieve antidepressant effects through the PI3K-Akt signaling pathway, the relaxin signaling pathway, the MAPK signaling pathway, the endocrine resistance, the AGE-RAGE signaling pathway in diabetic complications, the HIF-1 signaling pathway, the dopaminergic synapse, the estrogen signaling pathway, the Focal adhesion, the cAMP signaling pathway, the TNF signaling pathway, the Ras signaling pathway, the Neuroactive ligand-receptor interaction, and so on.

## 4. Discussion

The network pharmacology results showed that, with conditions of OB ≥30% and DL ≥0.18, 14 main active components of XPJYD were identified, and these components were predicted to target 45 potential depression-related proteins. Four important active components of XPJYF were identified from the drug-component-target-disease network, namely, quercetin, luteolin, kaempferol, and beta sitosterol. Quercetin, which can improve the level of monoamine transmitters in the brain and regulate the activity of the HPA axis, plays an important role in the treatment of depression [61]. Luteolin may play an antidepressant role by inhibiting endoplasmic reticulum stress-induced apoptosis [62]. Kaempferol, which has antioxidant and anti-inflammatory properties, may exert its antidepressant effects by increasing the Akt/*β*-catenin cascade in the prefrontal cortex [63]. In addition, kaempferol reduced the concentrations of inflammatory mediators IL-1*β* and TNF*α* in the prefrontal cortex of depressed mice, especially at a dose of 20 mg/kg [64]. Beta-sitosterol shortened the immobility time of depressed mice in forced swimming experiments and regulated the levels of NE, 5-HT and the metabolite 5-HIAA in the brains of mice. Its antidepressant effect was similar to that of the positive control, 30 mg/kg fluoxetine [65]. Relevant literature has reported that these active ingredients are significantly associated with depression, suggesting that XPJYD may have an antidepressant effect through these active ingredients.

PPI network analysis showed that VEGFA, EGFR, CASP3, IL6, ESR1, and other targets have important implications in the treatment of depression with XPJYD. Studies have shown that AKT1, VEGFA, ESR1, IL6, and AR play an important role in the treatment of depression [47–49, 53, 55, 59, 60]. An experimental study showed that XPJYD reduced the serum and hippocampal levels of IL-6, TNF*α,* and other inflammatory factors in depression model rats and improved their learning and memory behaviors [66]. The molecular docking results showed that beta sitosterol had good docking results beta sitosterol with AR, FOS, AKT1, VEGFA, NR3C1, and NOS3 genes and formed hydrogen bonds with FOS, VEGFA, NR3C1, and NOS3 genes, suggesting that beta sitosterol may be the main active ingredient of XPJYD involved in its antidepressant effect.

**Figure 8:**
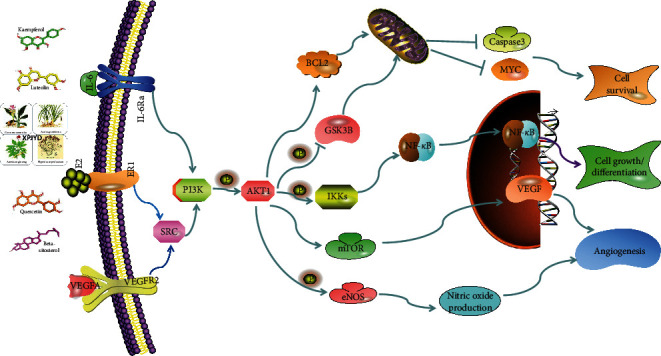
XPJYD regulates the PI3K-Akt signaling pathway.

GO function analysis shows that XPJYD is involved in biological processes that can achieve antidepressant effects, such as apoptotic signaling pathway, cellular response to lipid, inflammatory response, neuron death, response to reactive oxygen species, and others. KEGG pathway analysis showed that XPJYD mainly regulates 13 pathways such as PI3K-Akt signaling pathway and estrogen signaling pathway to treat depression. The PI3k-Akt pathway, a classic antiapoptotic and prosurvival pathway, is widely found in various cells and has biological functions that include regulating cell growth, proliferation, differentiation, migration, and autophagy [67]. In nerve cells, the PI3K-Akt pathway not only regulates the proliferation and differentiation of nerve cells and induces the autophagy of nerve cells but also participates in regulating blood flow in the brain and promoting the survival of neurons, which is closely related to the occurrence and development of depression [68, 69]. Studies have shown that the occurrence of depression is associated with key targets in the PI3K-Akt pathway, which is also one of the pathways in which many antidepressants exert important effects. Venlafaxine is a nonclassical antidepressant that inhibits the reuptake of the neurotransmitters 5-HT and NA. Venlafaxine can affect the PI3K-Akt pathway to inhibit the apoptosis of hippocampal neurons and improve the behavior of depressed rats [70]. A study has found that XPJYD inhibited the expression levels of PI3K, Akt, and P-Akt, thereby regulating the PI3K-Akt pathway, protecting the vascular endothelium of CUMS-depressed rats, and improving depression symptoms in rats [71]. XPJYD may regulate cerebral blood flow and promote neuronal survival through the PI3K/Akt signaling pathway to treat depression, as shown in Figure 8. Studies have shown that XPJYD improved the serum VEGFA concentration, regulated the PI3K/Akt/eNOS-specific pathway, regulated cerebral cortex blood flow, and improved depression symptoms in rats with chronic unpredictable mild stress-induced depression [50].

The estrogen signaling pathway plays an important role in the treatment of depression. Some reports suggest that estrogen replacement therapy has antidepressant effects in both clinical and animal studies [72, 73]. Studies have shown that estrogen can increase the 5-HT content in serum by inhibiting the degradation of monoamine oxidase and promoting an increase in tryptophan hydroxylase levels [74, 75]. Estrogen not only regulates gene transcription but also binds to estrogen receptors on cell membranes to activate the PI3K signaling pathway [76]. XPJYD, which may have activity similar to that of estrogen, could increase the 5-HT content in the brain by estrogen receptors to affect the PI3K signaling pathway. Literature studies have found that the occurrence, development, and treatment of depression are related to relaxin signaling pathway [77], MAPK signaling pathway [78], endocrine resistance [79], AGE-RAGE signaling pathway in diabetic complications [80], HIF-1 signaling pathway [81], dopaminergic synapse [82], focal adhesion [83], cAMP signaling pathway [84], TNF signaling pathway [85], Ras signaling pathway [86], and neuroactive ligand-receptor interaction [87]. Thus, we infer that the antidepressant mechanism of XPJYD is realized through the regulation of the above 13 pathways, which remained to be further validated. In addition, pathway enrichment analysis showed that some tumor-related pathways may be related to the antidepressant mechanism of XPJYD. In later research stages, we will further explore related pathways.

Preliminary experimental results showed that XPJYD may increase the content of 5-HT and DA in the brain, improve cerebral blood flow, and inhibit the inflammatory response and HPA axis hyperactivity. The network pharmacological results showed that XPJYD may regulate 13 pathways such as the PI3K-Akt signaling pathway and estrogen signaling pathway to treat depression. Most network pharmacology studies are based on virtual screening of databases, while TCM compounds can only show a therapeutic role on the basis of in vivo processes such as oral absorption, distribution, metabolism, and excretion. Due to the limitations of network pharmacology, this study needs further experimental verification.

## 5. Conclusion

In summary, network pharmacological analysis revealed characteristics of the mechanism of action by which XPJYD treats depression, which involves multiple components, multiple targets, and multiple pathways. XPJYD is an important modulator of AKT1, VEGFA, ESR1, IL6, and AR through active ingredients such as beta sitosterol. XPJYD achieves antidepressant effects by regulating 13 pathways, including the PI3K-Akt signaling pathway and estrogen signaling pathway. Although these 13 pathways have been shown to be strongly associated with depression, some pathways have not yet been shown to be regulated by XPJYD. Therefore, these pathways provide experimental research directions for the further study of XPJYD in the treatment of depression.

## Figures and Tables

**Table 1:** Potential active ingredients of XPJYD.

	Mol ID	Ingredient	OB	DL	Drug
1	MOL000359	Sitosterol	36.91	0.75	YJ, GYJST
2	MOL004328	Naringenin	59.29	0.21	YJ
3	MOL000358	Beta-sitosterol	36.91	0.75	YJ, XYS
4	MOL006980	Papaverine	64.04	0.38	XYS
5	MOL006774	Stigmast-7-enol	37.42	0.75	XYS
6	MOL005344	Ginsenoside rh2	36.32	0.56	XYS
7	MOL011455	20-Hexadecanoylingenol	32.7	0.65	XYS
8	MOL008397	Daturilin	50.37	0.77	XYS
9	MOL008173	daucosterol_qt	36.91	0.75	XYS
10	MOL011442	(8S,9S,10R,13R,14S,17R)-17-[(1R,4R)-4-Ethyl-1,5-dimethylhexyl]-10,13-dimethyl-1,2,8,9,11,12,14,15,16,17-decahydrocyclopenta[a]phenanthren-7-one	43.87	0.75	XYS
11	MOL000422	Kaempferol	41.88	0.24	SCP, GYJST
12	MOL003578	Cycloartenol	38.69	0.78	SCP
13	MOL003576	(1R,3aS,4R,6aS)-1,4-Bis(3,4-dimethoxyphenyl)-1,3,3a,4,6,6a-hexahydrofuro[4,3-c]furan	52.35	0.62	SCP
14	MOL003542	8-Isopentenyl-kaempferol	38.04	0.39	SCP
15	MOL000098	Quercetin	46.43	0.28	GYJST
16	MOL000073	Ent-epicatechin	48.96	0.24	GYJST
17	MOL000006	Luteolin	36.16	0.25	GYJST
18	MOL000492	(+)-Catechin	54.83	0.24	GYJST

## Data Availability

The data used to support the findings of this study are included within the article and supplementary information files.

## Supplementary Materials

Supplementary Materials Figure 6 data: GO enrichment analysis data for the overlapping targets needed to draw Figure 6. Figure 7 data: KEGG pathway enrichment data for the overlapping targets needed to draw Figure 7.
