# Supplementary material for: Therapeutic Targets and Mechanism of Xingpi Jieyu Decoction in Depression: A Network Pharmacology Study
Source: Evid Based Complement Alternat Med. 2021 Jun 23;2021:5516525. doi: 10.1155/2021/5516525 (PMC8249129; doi:10.1155/2021/5516525)
Supplement: Supplementary Materials — Figure 6 data: GO enrichment analysis data for the overlapping targets needed to draw Figure 6. Figure 7 data: KEGG pathway enrichment data for the overlapping targets needed to draw Figure 7. [file 5516525.f1.zip › 5516525.f1/SUPPLEMENTARY DESCRIPTION.doc]

**SUPPLEMENTARY DESCRIPTION**

**Supplementary Material**

**Figure 6 data**: GO enrichment analysis data for the overlapping targets needed to draw Figure6.

**Figure 7 data**: KEGG pathway enrichment data for the overlapping targets needed to draw Figure7.
